# The Experience of Women Receiving Mastectomy Care in an Acute Surgical Ward: A Qualitative Study

**DOI:** 10.1002/nop2.70053

**Published:** 2024-10-07

**Authors:** Celine Frost, Raewyn Lesā, Sandra Richardson

**Affiliations:** ^1^ Centre for Postgraduate Nursing Studies University of Otago Christchurch New Zealand

**Keywords:** breast cancer, inpatient experience hospital experience, mastectomy

## Abstract

**Aim:**

The research aim was to understand the inpatient experience of women who received postoperative care for mastectomy surgery in an acute surgical ward.

**Design:**

A Qualitative Descriptive Research design was used.

**Methods:**

The lead researcher conducted individual semi‐structured interviews with 10 women who received postoperative care following mastectomy surgery in an acute hospital surgical ward. The transcribed interview data was analysed using a thematic analysis.

**Results:**

A mastectomy is a momentous event in any woman's life. In the initial post‐operative phase, the physical intrusion of surgery, aesthetics of the ward, and mixed‐gender rooms were all found to influence the women's journey towards holistic recovery. Therapeutic care from all in the healthcare team and a ward setting conducive to recovery were identified as favourable influences towards physical and emotional healing.

**Conclusion:**

Recognising that a mastectomy ‘is not just physical’ may help the breast care team optimise the first 24 h of postoperative care in preparation for the challenges the women may face on discharge.

**Implications for the Profession and/or Patient Care:**

In an acute inpatient setting, a women's physical needs may be prioritised over the psychological and emotional ramifications of a mastectomy. Post‐operative inpatient care for breast cancer surgery requires not only professional competency with clinical and technical tasks, but therapeutic nursing and communication skills to support women's psychological needs.

**Patient or Public Contribution:**

The findings were drawn from 10 women, narrating their inpatient experience for breast cancer surgery. All women had undergone a unilateral or bilateral mastectomy following breast cancer.

## 1 Introduction

Breast cancer remains one of the most prevalent forms of cancer for females. The World Health Organisation (WHO) identified it as the commonest cancer in women in 157 of 185 countries in 2022, causing 670,000 deaths globally (WHO 2024). In New Zealand, around 3000 women are diagnosed with breast cancer each year (Campbell et al. 2018). For localised breast cancer, a simple or total, unilateral, or bilateral mastectomy (surgical amputation of partial or entire breast tissue with or without the nipple) is the primary treatment (Campbell et al. 2018).

Breast cancer differs from most other diseases due to its connection to a symbolic female organ (Hofmann 2021). In many cultures, the breasts are a socially accepted symbol of womanhood; representing the feminine body, beauty, sexuality and fertility and nurturing as a mother (Hofmann 2021; Zeighami Mohammadi, Mohammad Khan, and Zohreh Vanaki 2018). Therefore, losing the breast(s) is often a devastating, emotional experience for a woman as she learns to adjust and cope with the distortion between the self and the physical body which can create feelings of confusion, strain and uncertainty (Paraskeva et al. 2019; Urio et al. 2019).

In New Zealand (NZ), the initial post‐operative nursing care for women undergoing a mastectomy is usually provided by general surgical nurses during a 1‐ to 2‐day hospital stay in an acute surgical ward (Breast Cancer Foundation NZ 2024). Examples of this nursing care include monitoring vital signs, inspecting suture line(s) and wound drain(s), and administering analgesia and anti‐emetics. Most patients can perform self‐care independently or with minimal assistance a few hours post‐anaesthetic (usually in relation to age and extensiveness of the operation). Managing complex emotions in a busy postoperative ward may be challenging for some women. The aim of this qualitative study was to explore the inpatient experience of women in an acute care setting after having a mastectomy for breast cancer. The purpose was to generate data to inform holistic models of care for breast cancer teams who provide care to women post‐mastectomy in the inpatient setting.

## 2 Background

The psychological and physiological experiences of women post‐mastectomy have been greatly researched, however, most studies have focused on post‐discharge from hospital (6 months to 15 years post‐surgery) rather than their immediate post‐surgery inpatient experiences (Hofmann 2021; Paraskeva et al. 2019; Urio et al. 2019; van Ee et al. 2019; Zeighami Mohammadi, Mohammad Khan, and Zohreh Vanaki 2018). In several of these studies, the inpatient experience has been identified as a key touch point or theme, despite the research being conducted in a timeframe that means women may have adjusted to their altered body and lifestyle (Hofmann 2021).

In the initial recovery period, gratitude for survival is more important than aesthetics for many women. While some may view their altered appearance as a symbol of survivorship, others, have spoken about a loss of status in the family, changes in partner intimacy and sexual relationships, and abstinence from social interactions due to feelings of deformation, unattractiveness and/or inferiority (Hofmann 2021; Phoosuwan and Lundberg 2023). Some women have talked about feeling their identify was robbed (Zeighami Mohammadi, Mohammad Khan, and Zohreh Vanaki 2018).

Observing the altered appearance of their chest post‐operation is often a momentous time in a mastectomy patient's recovery (Herring et al. 2019). The results from one study that surveyed 128 women about the experience of looking at their mastectomy and/or reconstruction identified most had been worried about what they would see (*n* = 94, 75%). However, 34 participants (27%) were keen to see the post‐surgical outcome, while 37 (30%) initially avoided looking at their breast area (Paraskeva et al. 2019). Considerable amount of physical and psychological support was therefore required; yet 63% of the participants in this study were alone when they first viewed their post‐operative appearance in the ward environment (Paraskeva et al. 2019). Researchers have raised concerns that nurses may be inadequately prepared to support women for the first viewing of their post‐operative wound(s) (Herring et al. 2019).

The challenges associated with an altered self‐perception and changed social environment mean women undergoing mastectomy often need extra psychosocial support (Carr et al. 2019). However, in the acute care setting, a patient's physical needs may be prioritised over their psychosocial needs due to busy workloads and time constraints. Consequently, patients may feel the effect of nurses being torn between holistic models of nursing care and the task‐orientated nature of the biomedical model which the health system demands (Culbertson et al. 2020). This study sought to understand the inpatient experience of women who received postoperative care for mastectomy surgery in an acute surgical ward. Capturing the experience of these women offers insights about how to best optimise the first 24 h of postoperative care to prepare women for the emotional and psychological challenges they may face on discharge.

## 3 Methods

### 3.1 Study Design

A qualitative descriptive design based on in‐depth, semi‐structured interviews was adopted to give women a voice and present rich descriptions that do not stray far from the participants' voices (Doyle et al. 2020). The research question addressed was ‘how do women undergoing a mastectomy experience their inpatient stay in an acute setting?’.

### 3.2 Setting and Sample

The study setting was an acute surgical ward in a public hospital in NZ. Accommodated specialities included general surgery, plastic surgery, orthopaedic surgery and occasionally, general medicine. The 30‐bed ward was configured as four, six‐bedded rooms and six single rooms. Some rooms offered views of natural scenery and others looked out onto a concrete exterior. Where possible, the mastectomy patients shared a six‐bedded room with other women although occasionally, male patients were also placed in the room due to bed shortages. The length of inpatient stay for mastectomy patients is typically one to two nights and each patient has the same surgeon throughout their treatment. Their assigned nurse varies depending on rosters although mastectomy patients also receive support from a specialist breast care nurse (BCN), who assists in the coordination of care and information delivery.

### 3.3 Recruitment

Sampling of participants was purposeful to enable the gathering of rich accounts of the experience of inpatient post‐mastectomy patients. Potential participants were females aged 18 or above who had undergone unilateral or bilateral mastectomy surgery for breast cancer and received postoperative care on the specified surgical ward until discharged. Women who had prophylactic mastectomy surgery due to carrying the breast cancer gene 1 or gene 2 mutation or had immediate reconstruction surgery were excluded. To recruit participants, the lead researcher discussed the study with the specialist breast care nurses in the hospital who agreed to provide a brief information sheet to women who met the criteria during the patient's outpatient appointments (4 weeks pre‐surgery, day of hospital discharge and one‐week post‐surgery). Flyers advertising the study were also displayed in the waiting areas of the breast cancer clinic and the general surgery outpatient department. To raise awareness, the study was discussed at a ward meeting in the lead researcher's workplace.

The information sheet provided the lead researcher's contact details and potential participants were asked to contact her to raise queries and/or express interest in participating. Participants who expressed interest were emailed or given physically, comprehensive information about the study, a consent form and a prepaid envelope, addressed to the lead researcher. The lead researcher then made contact to schedule the interview. The aim was to recruit a maximum of 10 participants to allow for a more in‐depth and intensive analysis of each individual's experiences and thus answer the research question. The researcher continued to recruit until this number was reached. Recruitment commenced August 2018 and the final participant was recruited in April 2019.

### 3.4 Data Collection

To capture detailed accounts of the women's experiences, the lead researcher developed open‐ended questions focusing on the participants' inpatient experiences post‐mastectomy. These questions were then practised among the research team to enhance clarity. Demographic information was also collected from each participant (see Table 1). To enable a deeper exploration of the participants responses, the questions were used as a guide only. The lead researcher facilitated and audio‐recorded each interview which ranged in duration from 35 min to 2 h. The length of each interview was determined by the depth of exploration required, as guided by the participants' responses. At their preference, all interviews took place in the participants' homes.

**TABLE 1:** Interview guide.

Interview Guide
**Demographics** Which of the following best represents your age group? 18–24; 25–29; 30–34; 35–39; 40–44; 45–49; 50–54; 55–59; 60–64; 65–69; 70–74; 75–79; 80–84; 85. Which of the following best represents your ethnicity? (You may select more than one) NZ European; NZ Maori; Pasifika; Asian; Other European; Middle Eastern/Latin American/African. Other (please specify) _______________________
**Questions** What do you remember most about your time in hospital? Who looked after you when you were in hospital? What were their roles? How often did you see them during your time in hospital? What was the best part of your hospital experience? What was the worst part of your hospital experience? Which hospital staff do you remember and why? Can you tell me about your experiences of your right to privacy. Were you able to ask for help when you needed it and if so, did you ask? What do you remember most about the room you stayed in? Were there things you liked or disliked? What were your experiences of receiving discharge information on the day you left the hospital? Do you have any specific spiritual or cultural practices or beliefs and were these acknowledged and/or met? If so, can you give examples? How well do you feel your emotional needs were recognised? Is there anything you wish that had been done differently?

### 3.5 Data Analysis

The interview transcripts were analysed using Braun and Clarke's (2021) six steps of thematic data analysis. The aim was to identify, analyse and document patterns, meanings, and themes from the data. Analysis commenced with the lead researcher repeatedly reading each transcript and noting interesting or significant points in the margins. The initial codes assigned to each transcript represented key words or expressions from across the data set and were derived from the meaning the lead researcher ascribed to the text. These codes were manually underlined with unique colours assigned to each code. A colour‐coded key was created for each transcript and used to compare information across transcripts. From the coded data, main codes were selected to construct themes for further exploration. These themes were generated through inductive analysis, derived directly from the raw data.

By the sixth interview, the lead researcher noted similar ideas were emerging signifying the achievement of informational redundancy. The final four transcripts suggested data saturation. While these transcripts did not produce new codes, they highlighted points of interest and expanded the already established codes. The final themes were generated inductively by categorising the codes according to commonality in meanings, possible hierarchies and/or contradictory points of view. All three researchers regularly liaised to determine codes and agree on final representation of the themes. Areas of initial discrepancy were deliberated until mutual agreement was reached. Table 2 shows an example of theme identification.

**TABLE 2:** Example of theme identification.

Initial codes	Categorisation of codes	Theme and its definition
Viewing scar for first time. Shock at seeing the disfigurement. First post‐surgery shower. Loss of femininity. Life factors. Women's attitude ‘Elective’ surgery suggested choice. ‘Simple mastectomy’ may underplay the seriousness.	Challenges associated with an altered body image and disfigurement. Conflicting emotions. Readiness to view the scar. Mental, psychological, and physical. Impact of life factors and attitude. Use of clinical terminology.	**It's not just physical.** Participants struggled with conflicting emotions whilst coming to terms with their mastectomy. The dramatic impact on physical body image brought threats to femininity and feelings of disfigurement including changes in sexual identity, confidence in appearance and intimate aspects of womanhood. A gradual introduction to viewing the full scar may be beneficial as well as considering clinical terminology.
Task focused. Therapeutic care. Presence and reassurance Independence or assistance. Reluctant to use call bell. Want to feel normal. Time to talk	Task verses therapeutic Initial post‐operative phase challenging. Present, kind and reassuring nurses helpful. Sometimes needed independence, sometimes assistance. Reluctant to ask for help.	**Therapeutic Care** While the participants acknowledged clinical tasks were important, they also wanted an understanding of the emotional aspects of the surgery, defined as therapeutic care. Several participants spoke about the differences in task focused and therapeutic care.
Busy, noisy environment. Many disruptions. Very little privacy. Not conductive to rest. Rapport with others. Inadequate coverage. Potential body exposure. Open light room p Unkempt exterior	Feelings of comradery. Sense of community. Vulnerability when rooming with males. Room aesthetics may impact recovery.	**An Environment Conducive to Recovery** The reality of a hospital ward is that it is often busy and noisy e with many disruptions. Privacy was particularly challenging for the participants because intimate discussions regarding their care were not always confidential. Women may feel vulnerability when rooming with males. Room aesthetics is important.

### 3.6 Ethical Considerations

The interviews were recorded on a digital voice recorder, transferred to a password protected computer and then deleted from the device. Pseudonyms are used in the reporting of this research. Participants were given the option of a support person for the interview which no participants took up. They were also reminded that they could decline to answer any questions or ask to pause the interview should they feel upset. During three interviews, the lead researcher noted some participant discomfort and paused the interview. All participants wished to continue after a few minutes. Ethics approval was granted by the University (reference H18/052) and locality approval attained from the hospital where the study took place.

Consultation with representatives of the indigenous population, New Zealand Māori, was undertaken as part of the research ethics process. None of the participants in this study identified as Māori, based on self‐disclosure of ethnicity at the time of recruitment.

### 3.7 Trustworthiness and Credibility

The research team included two PhD qualified nurses with extensive experience in qualitative research approaches, including the use of interview methods and thematic analysis. To manage the risk of insider knowledge as a potential research bias, reflexivity was carried out prior to, and for the duration of the research (Holmes 2020). The researcher understood that this study was informed by her clinical experiences as a RN who worked closely with women post‐mastectomy for breast cancer and acknowledged the influence of personal values, experiences from the role, knowledge and preconceived ideas may have on the research process and ultimately the results. Additionally, during data analysis, all three researchers reviewed, codes and themes together, remaining open to alternate meanings of the text. To enhance credibility of the findings, the final themes are supported by verbatim participant quotes.

## 4 Findings

The findings depict the voice of 10 women, narrating the breast cancer journey and initial inpatient experience. Ethnicity and age of the women are presented in Table 3.

**TABLE 3:** Age and ethnicity of the participants.

Name (Pseudonym)	Age (Years)	Ethnicity	‘Other’ Ethnicity
Alice	55–59	New Zealand (NZ) European	
Bridget	50–54	NZ European	
Chloe	65–69	NZ European	
Dora	40–44	Other European	
Emma	75–79	NZ European	
Fran	40–44	NZ European	
Gloria	70–74	NZ European	
Hetty	50–54	NZ European	Canadian
Ivy	85+	NZ European	English
Joan	60–64	Other European	

The women were unanimous that a mastectomy is a significant event, and the experience is not just physical. Therapeutic care from all in the healthcare team and a ward setting conducive to recovery were more favourable to healing both physically and emotionally. These findings are presented in three themes.

### 4.1 Theme 1 – It's Not Just Physical

The essence of this theme is that the physical removal of a breast may impact women on psychological, social and representational levels. Many participants experienced feelings of disfigurement and a loss of femininity, prompting questions about the breast's significance, including its symbolism to motherhood and its role in an altered family status.

For the participants, the response following a mastectomy was not just about the physical loss of a body part but also the challenges associated with an altered body image. Although the loss of the breast was accepted as necessary, the modified physical form of femininity presented challenges to the participants in relation to a loss of self and sexuality. As Hetty explained, her breasts were intrinsically linked to who she was as a woman:

It is part of you … I was never an exhibitionist but it's part of being a woman, right? Hetty

All participants dealt with conflicting emotions. Some struggled with what the physical changes would look like, while others did not feel the impact until after the surgery. Hetty said she tried to prepare herself pre‐operatively by viewing a photograph on the internet of a woman with a post‐mastectomy scar, yet after the surgery, she cried during the surgeon's visit:

It took me five weeks to look at an image on the internet… Once I saw it online it was easier for me to come to terms with what was going to happen to me … When I saw my surgeon I cried, because it was all reality … prior to the operation it wasn't. Hetty

Joan had previously undergone a lumpectomy for breast cancer and said the first time she was scared, but this time she was *more angry*. For Dora, her scar was a symbol of the breast cancer journey; visible evidence it may have saved her life.

When I saw [for] the first time…my scar [I] was like, oh, shock for my system but on the other hand [I] felt the relief because the main threat for my life was gone…so I can live with a visual scar. Dora

One of them said I've only got one breast now and I said, ‘well I look at it that the cancer was in the breast and now it's gone’. Joan

Many were apprehensive and challenged by conflicting emotions towards seeing their altered appearance for the first time.

I didn't like what I see … to be honest…it's like a reminder forever of what happened to me but it's also a good reminder [of] the people who took care of me. Dora

[The nurse] said “oh what a lovely job”, so I had a good look to see the good job. I wasn't frightened and I wasn't upset … I just accepted it because it's cured me. Ivy

The first shower post‐surgery was a significant moment during the participants inpatient experience, particularly in relation to a readiness to view the scar.

I was going to have a shower and they said you can have extra towels to cover it if you don't want to look… I think possibly because I said I don't want to look just yet. Hetty

Alice described this shower as a *mental, psychological and physical* experience. Although the importance of this moment was recognised by some nurses, others only provided for the participants physical needs.

It would have been so much easier and better if the nurse had said, ‘I'll come with you for the first shower’ and not because I was just a bit unsteady but just the emotional and that first moment of showering when you're minus a breast. Alice

A gradual introduction to viewing the full extent of the scar(s) was found to be beneficial for some participants. This approach consisted of two phases, first looking directly down at the scar and second, seeing a full‐frontal view. Bridget intentionally chose to view the scar this way whereas the two‐phased approach was incidental for Joan and Hetty.

It was nice to just go into the washroom by myself and take my gown off and just look … when you're looking down, changing dressings and things, you don't really see the whole chest area it's just from the top. Bridget

[A nurse] said “have you seen it yet?” and I went “no.” Then one day I did by accident, and I thought…that was a bit of a surprise… I'm not actually ready to have a full‐frontal view just yet…When I went to have a shower, … there wasn't a mirror and I thought ‘that's good I don't have to [look at it yet]’. Joan

What was really good was that the mirror stopped there [at the décolletage] so when I looked at myself without anything on, I didn't see it [the wound]. So, it was still me. Hetty

The decision to undertake this form of treatment was hard in itself. Life factors (age, marital or relationship status, motherhood, previous exposure to breast cancer, occupation, and attitudes) influenced the participants' rationale for agreeing to surgery, and their ability to come to terms with the mastectomy and cope post‐operatively. Previous experiences with breast cancer in themselves, or with friends, provided comfort for some participants as they understood the process and the expected recovery timeline. Ivy chose a mastectomy (as opposed to prolonged treatment options) because her husband was elderly and parking difficulties at the hospital would have added to her stress. Gloria welcomed her hospital stay as it provided relief from her domestic duties which included supporting her elderly mother. For Emma, being single, meant she could focus on recovery. She also felt that being older influenced her outlook on her breast cancer diagnosis.

I don't have to cook meals for a family… I can sit down if I don't feel too good … I can go outside if I feel I want to… You have a longer‐term view of what's important in your life and small things don't bother you so much … you've had a few knocks and whatever anybody's experienced in their life has shaped how they look at things. Emma

Some participants spoke about the clinical terminology used during their hospital stay and its impact. For example, Bridget said the word ‘elective’ surgery suggested she had a choice, of which she felt there was none because she was advised that a bilateral mastectomy was the best treatment. Ivy thought the words ‘simple mastectomy’ underplayed the seriousness of the procedure.

Nobody stressed how serious it was … in the report, … it's just down as a simple mastectomy. That's what struck me, it was ‘simple’, and I thought, what's a complicated one? Ivy

Elective's a funny word really … I remember the pre‐admission form; the first question was do you still want to have your surgery? I thought no jeepers I don't, I would never have chosen to do this, but yes, I know I need it. Bridget

### 4.2 Theme 2 – Therapeutic Care

On return to the ward, nurses observe their patients, monitor vital signs, inspect the surgical site and drains, administer medications as required and assist with personal cares. While the participants acknowledged the importance of these clinical tasks, they also wanted an understanding of the emotional aspects of the surgery, defined as therapeutic care. Several participants spoke about the differences in task focused and therapeutic care, captured in these examples from Alice.

Nurses [would] just check the wound as it if was just another surgery or wound site…There was no recognition of the surgery that I'd had… It was just more checking the wound, checking the obs [sic] and ‘did I need anything for pain?’ Alice

She wasn't there just to take my temperature and blood pressure and that sort of thing … she just had a little bit more time just to have a chat to me about some of my concerns…she just seemed a little bit more intuitive to my needs. Alice

The participants described the initial post‐operative phase as the most challenging, requiring a sense of security and nursing sensitivity. Nursing support in the form of being present, kind words and reassurance that ‘not feeling ok’ was a natural part of the post‐operative journey was seen as beneficial to the emotional aspects of recovery.

What is needed is compassion and just acknowledgement that it's a ‘shitty’ time and it will get better, and we're allowed to just not feel 100% the day after we've just lost a symbol of womanhood. Hetty

Some participants said they wanted the nurse to recognise when they needed assistance or independence, both emotionally and physically. Emma said she wanted to shower independently so she could test her readiness for discharge.

She stood outside the door and said “give me a shout when you're ready” …she would have come in and done it fo12r me, but I said “no, I can do it myself” … I just wanted to see how much I could do for myself because I knew I was going to have to do it at home. Emma

Other participants said they did not receive assistance with their personal cares unless they specifically requested help which was challenging for those with physical limitations. Some said they were reluctant to use the call bell, for fear of adding to the nurses' busy workloads or disturbing their routine duties or ‘cup of tea break’.

I knew there was all this pressure and I thought, ‘no you can't be asking’. Ivy

I think I pressed it [bell] twice my whole stay… why would you? they're busy enough. Fran

The participants appreciated nurses chatting to them while doing their tasks. Fran spoke about a nurse who *stuck out* because although busy, she talked as she worked. Dora said she was treated as a person which made her feel she was a *normal person*. Alice appreciated the nurse who cared for her consecutively over 3 days as she *actually took the time* to talk with her which was what she needed.

### 4.3 Theme 3 – An Environment Conducive to Recovery

Ideally, the ward is a place of recuperation yet in reality, it is often a busy, noisy environment with many disruptions. Participants spoke of security issues, abuse towards staff and limited privacy. Privacy was particularly challenging for the participants because intimate discussions regarding their care may be overheard. Room allocation was subject to bed availability and the female to male ratio in the ward at a given time. Therefore, women undergoing a mastectomy could be rooming with male patients resulting in feelings of vulnerability for some participants. As a result, many participants believed the environment was not conducive to rest.

It was quite noisy for a start… I remember the noise of the other patients, particularly the lady opposite who seemed to eat chips all night even with my ear plugs in, and her phone she was receiving text. Alice

Several participants spoke about their assigned room. For many, rooming with other women who had undergone mastectomy was therapeutic as they could share their illness experience. Hearing other stories gave some participants confidence that post‐operative complications could be treated. Fran explained that while she usually refrained from *girly gossip, or womanly discussion* conversing with other women who had breast surgery was the most valuable part of her hospital experience. Other participants said they used humour as an antidote to negativity which lessened feelings of isolation. Feelings of comradery meant some intentionally left the curtains around their beds open:

We all kept our curtains open, even at night … not the first night so much because everyone was probably tired after the surgery but the next two nights everyone had their curtains open. Gloria

However, other participants were less keen to share a room with other mastectomy patients. Ivy said listening to *tales of woe* could be *quite depressing*. Hetty explained that there is always *somebody better off … or worse off* and this might not be a *good combination*.

Alice shared a room with five other people with different surgeries, one of whom was a male. She explained that she viewed herself as an outcast and withdrew into herself by closing her curtains as she felt uncomfortable due to the personal nature of her surgery.

I would have felt more comfortable if I was in a room with other women or one or two others who had the same surgery … it just felt so public I just felt that mine [surgery] was just completely different mentally and emotionally… even to get up and go to the toilet, I knew that they would all know … I'd had a mastectomy…I just felt I was in the wrong room for what I had been through. Alice

All participants shared the view that male occupancy in the same room was inappropriate. Some said they were ‘shocked’ when they realised, they were rooming with male patients. They spoke about feelings of vulnerability due to potential body exposure during intimate cares such as mastectomy site checks, and conversations about their surgery. When men were in proximity, inadequate coverage when wearing a hospital gown could create feelings of anxiety and embarrassment.

He was sitting up in bed … it was embarrassing getting out of bed with my gown on. Ivy

You're not going to talk to the man who's lying beside you about your booby and he most probably don't want to talk to you if he's got prostate [cancer]. Fran

You do hear the doctors discussing your problems … I don't think I'd like men in the room when they're discussing my boobs and things to be fair. Chloe

Several participants spoke about the impact of room aesthetics on their recovery. Emma explained that her outside view of an unkempt courtyard garden and unwashed exteriors meant she just wanted to *get home and look at something nice*. In comparison, Dora said the atmosphere was peaceful because her bed was positioned by the window with natural light and fresh air. Bridget spoke about the positive effects of an open room.

The outside [hallway] curtains were pulled back, the sun was streaming in. It was peaceful and quiet and there was just me on my bed … sitting there reading my book in this open space … when it's chocka… and the curtains are all pulled, it feels really claustrophobic and not very relaxing. Bridget

## 5 Discussion

This research explored the inpatient experience of women in an acute care setting after having a mastectomy for breast cancer. The findings highlight that after a mastectomy, facing the reality of an altered ‘self’ and female identity is challenging for many women. In the western ‘patriarchal culture’, the breast(s) are objects of attraction and sexual desire, thus from adolescence, many women feel judged by the appearance of their breasts (Hofmann 2021, p. 3). With the existence of pervasive body ideals, women may experience threats to their physical beauty and body image as the appearance of breasts change through puberty, childbearing, aging, or disease thus no longer aligning with societal norms or self‐expectation. Bodily imperfections particularly in relation to femininity are often hidden to avoid stigma, a reflection of cultural influence in western society (Hofmann 2021). In a metaphorical sense, women in this present study likened the removal of a breast to that of a soldier losing a limb in military action, in that both surgeries alter body image. Yet while there is potential for psychological harm in both situations, emotional trauma associated with a mastectomy is not often discussed.

For some participants, the mastectomy scar was symbolic, visible evidence of a procedure that may have saved their life. A feeling of gratitude for survival competed with the challenges presented when viewing their disfiguration for the first time. Viewing the wound meant the women may need to confront their anxieties or fears, whereas for others, viewing the wound helped them face the reality of their altered body. Experiencing a range of emotions and possible trauma reflects other research that found observing their altered appearance was often a significant moment in a mastectomy patient's recovery (Herring et al. 2019).

Feeling ready to view their altered appearance was important for the participants in this research. Even if they did not personally feel the need to limit the visual exposure of their scar, they appreciated the offer of wound coverage. The position of the mirror above the wash basin was incidentally found to be beneficial for some participants as this meant they could choose whether to partially, or fully view the wound. It also gave them control over how much of the wound they saw and an opportunity to pace this viewing according to their emotional readiness. Other studies have reported therapeutic benefits of mirror‐therapy post‐mastectomy (Tyner and Freysteinson 2023), as well as for neurological disorders or amputations (Wang et al. 2021; Thieme et al. 2018) which suggests there could be benefits of routinely implementing incremental viewing of the altered appearance post mastectomy.

The physical issue of showering was challenging for the participants in this study as it involved managing drains, tubing and wound dressings. For some, it was not physical assistance from the nurse that was required but psychological assurance from a person‐centred approach. The physical act of wearing a brassiere (bra) which previously may have been a source of enhancing femininity, comfort, and confidence was missed by some of the women. Nurses recognising when they needed assistance or independence, both emotionally and physically was important for the women suggesting that a person‐ centred approach requires nurses to be intuitive towards the type of support a patient needs at any given time (Herring et al. 2019).

Although the participants in this research were satisfied with the treatment offered, aspects of a therapeutic approach were inconsistent. Some observed that the inherent pressures in a busy surgical environment resulted in a focus on clinical tasks and personal opinions as a form of support, rather than using therapeutic communication (active listening, observing body language and mannerisms). Some participants welcomed nurses who performed tasks efficiently as they saw this as a total focus on their job, whereas others desired more therapeutic interactions, emphasising the need for nurse to be intuitive to their patient's needs. This included expecting the nurse to be able to recognise when they needed quiet time alone to come to terms with the mastectomy or when they wanted to engage in conversation. Some researchers have made links with a busy work environment and lack of compassionate care or privacy (Valizadeh et al. 2018) which poses questions as to how nurses can provide person‐centred care for post‐mastectomy patients in hospitals that are endeavouring to economise shorter‐stay without compromise to quality.

Connections with a breast cancer community once discharged may support the physical, spiritual, and emotional aspects of recovery (van Ee et al. 2019). This present research found that these connections were also valuable in the inpatient setting, creating feelings of camaraderie and diminishing feelings of vulnerability related to the intimate nature of their postoperative care which has also been reported (van Ee et al. 2019). Friendships established in the safety and security of the hospital environment may for some women, be valuable once discharged.

Some participants in this research struggled to accept room sharing with males. Although the nurses respected their privacy by drawing the curtains, it did not shut out noise from other patients or the external environment. In similar institutions to the one used in this study, mixed‐gender wards are governed by availability and space to create single‐gender rooms. Due to the intimate nature of breast cancer, several participants in this present study felt vulnerable in a mixed room and suggested male patients may also be embarrassed due to audible discussions of their diagnosis, treatment, and care. Despite the significance of the impact of mixed rooms, mention of such was not found in any published literature regarding the inpatient experience of women post‐mastectomy.

The physical environment was found to have a psychological impact on the inpatient experience for many participants in this study. Looking out to unkempt gardens or concrete walls could evoke feelings of depression and a rush to return home. An external view of natural surroundings has been shown to increase patient's satisfaction with their care and reduce hospitalisations (Hesselink et al. 2020).

The following recommendations are suggested as an initial way forward to address the needs of women coming to terms with a mastectomy in an inpatient public hospital setting. These may well have wider relevance or trigger similar considerations in other settings or patient populations.

Dedicating attention and nursing time to women viewing their altered appearance for the first time, along with therapeutic communication may help reveal hidden anxieties and prompt a person‐centred approach. Women's readiness to look at the scar(s) will vary; therefore, the nurse should assess their readiness and prepare the woman for an emotional reaction such as surprise, shock, anger, sadness, or relief. Offering support for this moment or suggesting viewing at a later stage if the patient is not ready, is recommended.

A gradual introduction to the post‐operative scar(s) may assist with greater acceptance of an altered body image and a move towards healing of self. A ‘two‐phase approach’ to visualising the scar(s) is likely to be beneficial for some women. In the initial phase, the woman looks down at her scar. This may take a few hours or several days depending on the readiness of the individual. This phase prepares the woman for the shock and reality of seeing the altered body image following her surgery. The second phase would incorporate seeing a full‐frontal view so the changed appearance can be visualised in the context of the whole person. This two‐phase approach could be enhanced with the use of mirrors and consideration of where they are placed in the ward. This method of incremental viewing may ease the patient into accepting the surgical outcome and address potential psychological trauma after breast cancer surgery.

Evident in this study was the variability of the inpatient experience. Avoiding an assumption that all women feel negatively towards their mastectomy and altered appearance is therefore important. The mastectomy scar(s) may act as a physical reminder for some women that the cancer has been removed and serve as a symbol of strength and survivorship. For women struggling with a loss of feminine identity, the process of enhancing or reconstructing this may be strengthened by helping with personal hygiene and if desired, applying moisturisers, perfume, or make‐up (away from the wound region).

For privacy, and to encourage women to speak more freely about their intimate surgery to health professionals, and significant others, avoiding mixed gender rooms is recommended. The creation of an area designed for relaxation and sharing of experiences may provide a sense of normality and help create a sense of community. If a mixed gender room is necessary, offering the patient pyjama bottoms (as the surgery is above the waist) and asking whether they would prefer their curtains open or closed may lessen feelings of vulnerability and exposure. Discussions between the patient and health professionals in a private room (away from the bedspace) is strongly recommended.

## 6 Limitations

While the study was open to all women who met the criteria, given the small sample size and a self‐selection recruitment strategy, there was limited variation among the sample in terms of demographic factors. The opportunity to engage with a wider range, potentially representing a more inclusive sample of New Zealand women, for example those from differing ethnicities, cultural backgrounds or sexual identities would further strengthen the understanding of individual's experiences.

## 7 Future Research

A larger study sample including national recruitment may give greater representation of diverse personal characteristics such as age, socio‐economic and familial status, which may influence the inpatient experience following mastectomy. A more culturally and geographically diverse cohort would provide the opportunity to identify any discrepancies within the care needs of patients identifying with different cultures.

## 8 Conclusion

The findings in this study show that a mastectomy is a significant event in any woman's life. Physically, the removal of the breast(s) leaves a scar which visually announces the disfigurement; however, unseen are the psychological ramifications of an altered body form and potential loss of feminine identity. In an acute surgical ward, the women's physical needs may be prioritised over helping women come to terms with the psychological and emotional ramifications of a mastectomy. The aesthetics of the ward atmosphere, physical intrusion of the surgery and consequent mixed‐gender rooms or facilities, impact on the women's ongoing journey towards holistic recovery. Post‐operative inpatient care for breast cancer surgery requires not only professional competency with clinical and technical tasks, but therapeutic nursing and communication skills to support women's psychological needs.

Recognising that a mastectomy ‘is not just physical’ may help all in the breast care team optimise the first 24 h of postoperative care in preparation for the challenges the women may face on discharge.

## Author Contributions

Made substantial contributions to conception and design, or acquisition of data, or analysis and interpretation of data: C.F., R.L. Involved in drafting the manuscript or revising it critically for important intellectual content: C.F., R.L., S.R. Given final approval of the version to be published. Each author should have participated sufficiently in the work to take public responsibility for appropriate portions of the content: C.F., R.L., S.R. Agreed to be accountable for all aspects of the work in ensuring that questions related to the accuracy or integrity of any part of the work are appropriately investigated and resolved: C.F., R.L., S.R.

## Conflicts of Interest

The authors declare no conflicts of interest.

## Data Availability

The data that support the findings of this study are available from the corresponding author upon reasonable request.
